# Gigahertz free-space electro-optic modulators based on Mie resonances

**DOI:** 10.1038/s41467-022-30451-z

**Published:** 2022-06-06

**Authors:** Ileana-Cristina Benea-Chelmus, Sydney Mason, Maryna L. Meretska, Delwin L. Elder, Dmitry Kazakov, Amirhassan Shams-Ansari, Larry R. Dalton, Federico Capasso

**Affiliations:** 1 Harvard John A. Paulson School of Engineering and Applied Sciences, Harvard University, Cambridge, MA USA; 2 Hybrid Photonics Laboratory, École Polytechnique Fédérale de Lausanne (EPFL), Lausanne, Switzerland; 3 Harvard College, Cambridge, MA USA; 4 Department of Chemistry, University of WAshington, Seattle, WA USA

**Keywords:** Optoelectronic devices and components, Organic-inorganic nanostructures

## Abstract

Electro-optic modulators are essential for sensing, metrology and telecommunications. Most target fiber applications. Instead, metasurface-based architectures that modulate free-space light at gigahertz (GHz) speeds can boost flat optics technology by microwave electronics for active optics, diffractive computing or optoelectronic control. Current realizations are bulky or have low modulation efficiencies. Here, we demonstrate a hybrid silicon-organic metasurface platform that leverages Mie resonances for efficient electro-optic modulation at GHz speeds. We exploit quasi bound states in the continuum (BIC) that provide narrow linewidth (*Q* = 550 at ${\lambda }_{{{{{{{{\rm{res}}}}}}}}}=1594$ nm), light confinement to the non-linear material, tunability by design and voltage and GHz-speed electrodes. Key to the achieved modulation of $\frac{{{\Delta }}T}{{T}_{\max }}=67 \%$ are molecules with *r*_33_ = 100 pm/V and optical field optimization for low-loss. We demonstrate DC tuning of the resonant frequency of quasi-BIC by ${{\Delta }}{\lambda }_{{{{{{{{\rm{res}}}}}}}}}=$ 11 nm, surpassing its linewidth, and modulation up to 5 GHz (*f*_*EO*,−3*dB*_ = 3 GHz). Guided mode resonances tune by ${{\Delta }}{\lambda }_{{{{{{{{\rm{res}}}}}}}}}=$ 20 nm. Our hybrid platform may incorporate free-space nanostructures of any geometry or material, by application of the active layer post-fabrication.

## Abstract (web summary)

Active photonics in free space is important in computing, imaging and sensing. Here, hybrid silicon-organic nanoscale structures change the intensity of a free-space light beam by applied microwave signals at gigahertz speeds with a high efficiency.

## Introduction

Recently, photonic technologies have become promising to address the bottleneck for high-speed communication^1^ and high-performance computing^2,3^ instead of traditional all-electronic technologies. The next-generation photonic devices need to manipulate light at high speeds, and most demonstrations today target either fiber or on-chip applications. Alternatively, metasurfaces are ideally suited for applications that require a compact control of free-space beams of light^4,5^, but most of them are static. Amongst the available mechanisms that provide active control^6,7^, hybrid^8^ electro-optic structures that employ *χ*^(2)^ effects to modulate optical fields by electronic signals are superior to alternative techniques when it comes to speed: the control fields may reach from the microwaves into the terahertz^9^ and are applied via metallic electrodes^10,11^ or antenna structures^12^. Several material platforms are available today, including organic nonlinear molecules^13^, barium titanate^14^, and lithium niobate^15^, which profit from advances in molecular engineering^16^, growth^17^, fabrication and stability^18^. Typically, a low optical loss and a high modulation efficiency are key for a wide range of applications and are especially crucial for quantum applications^19,20^.

Ultrathin electro-optic modulators from sub-wavelength resonators are exquisite candidates in applications that require tailor-cut control over free-space light in a compact way, such as free-space optical communication links^21^, coherent laser ranging, active optical components^22^, high-speed spatial light modulators^23,24^ and active control of free-space emitters^25^. Flat optical components such as metasurfaces^26,27^ rely on sub-wavelength-sized nanostructures that change the properties of a beam that is incident from free space onto the metasurface, and are ideally suited to address spatial multiplexing needs beyond the single pixel. From the perspective of the employed modulation mechanism, several have been proposed for metasurfaces, including the immersion of metasurfaces in liquid crystals^28^, their co-integration with epsilon-near-zero materials, phase-change materials^29–31^, semiconductor heterostructures^32^ or a change in refractive index by pumping with femtosecond pulses^33^. However, amongst all these mechanisms, the large majority of metasurface modulators of visible or telecom light trade an efficient modulation for high modulation speeds and vice-versa. Consequently, only a few reach modulation speeds in the microwaves, which are crucial for time-sensitive applications. The demonstrated switching speeds typically reach a few kilohertz to a few megahertz. As an outstanding candidate, the electro-optic effect^34–37^ is compatible with high-speed modulation, but the current active metasurface features low modulation efficiencies. This is linked to the sub-wavelength size of typical metasurface elements, leading to an interaction region only a few hundred nanometers long, commensurate with the thickness of flat optics. Furthermore, wavelength-sized resonators have long been characterised by low-quality factors as a result of their small azimuthal modal order.

**Fig. 1:**
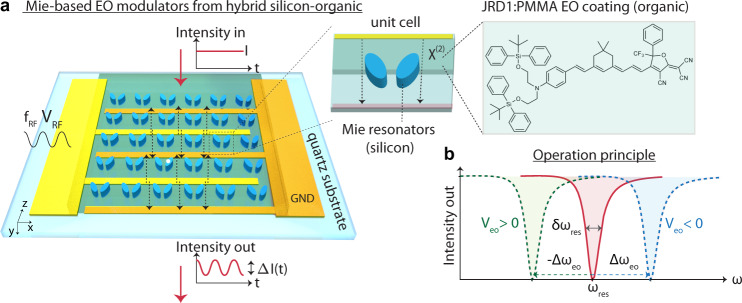
Ultrathin Mie-based free-space electro-optic modulators. **a** Free-space electro-optic modulators change the properties of a beam that is incident from free space. Sub-wavelength Mie resonators impart an intensity modulation to the incident light that propagates through the thin film via the electro-optic effect that occurs in an organic electro-optic coating that covers the nanoresonators; the interaction length is typically a few hundred nanometers long, shorter than a single wavelength, commensurate with the thickness of the organic electro-optic coating. **b** Resonant electro-optic modulators work on the principle that their resonant frequency ${\omega }_{{{{{{{{\rm{res}}}}}}}}}$ is tuned by Δ*ω*_eo_(*t*) linearly by an applied bias, due to the phase shift induced by the electro-optic effect. A radio-frequency bias *V*_RF_(*t*) = *V*_eo_ × *sin*(2*πf*_RF_*t*) is applied across two interdigitated electrodes (signal is shown in yellow and GND is shown in orange) displacing the resonance frequency around its zero bias value. Narrowband resonances that satisfy ${{\Delta }}{\omega }_{{{{{{{{\rm{eo}}}}}}}}} \, > \, \delta {\omega }_{{{{{{{{\rm{res}}}}}}}}}$ are preferred for full intensity modulation at low switching voltages. Dashed black arrows indicate the applied tuning field that introduces the electro-optic effect. Red arrows indicate the propagating optical field. EO electro-optic, GND ground.

In this work, we demonstrate efficient metasurface modulators that feature gigahertz tuning speeds by bringing together sub-wavelength resonators characterized by narrow linewidths, high-performance organic molecules, and a high-frequency electronic design into geometry as shown in Fig. 1a. In our design, a single layer of an organic nonlinear material (shown in green) is deposited on top of the sub-wavelength resonators. A microwave field is applied to the nonlinear material directly via metallic electrodes, which is advantageous, e.g., over optical pumping techniques with femtosecond pulses due to cross-compatibility with electronics. It changes the refractive index *n*_mat_ of the nonlinear material at optical frequencies via the linear electro-optic effect, also known as Pockels effect, by ${{\Delta }}n(t)=-\frac{1}{2}{n}_{{{{{{{{\rm{mat}}}}}}}}}^{3}rE(t)$, with *r* the electro-optic coefficient of the material and $E(t)=\frac{{V}_{{{{{{{{\rm{RF}}}}}}}}}(t)}{d}$ the tuning field (voltage *V*_RF_(*t*) applied across the distance *d*). In a resonant modulator that employs the *r*_33_ component of the electro-optic tensor, this change in refractive index Δ*n*(*t*) modifies its resonant frequency as illustrated in Fig. 1b by ${{\Delta }}{\omega }_{{{{{{{{\rm{eo}}}}}}}}}(t)=-\frac{{{\Delta }}n(t)}{{n}_{{{{{{{{\rm{mat}}}}}}}}}}{\omega }_{{{{{{{{\rm{res}}}}}}}}}{{{\Gamma }}}_{c}={g}_{{{{{{{{\rm{eo}}}}}}}}}{V}_{{{{{{{{\rm{RF}}}}}}}}}(t)$, with ${g}_{{{{{{{{\rm{eo}}}}}}}}}=\frac{1}{2}{n}_{{{{{{{{\rm{mat}}}}}}}}}^{2}{r}_{33}\frac{1\,V}{d}{\omega }_{{{{{{{{\rm{res}}}}}}}}}{{{\Gamma }}}_{c}$ the electro-optic coupling rate at 1 V applied voltage^38^ and Γ_*c*_ the overlap factor of the two interacting fields with the nonlinear medium. The shift is proportional to the applied voltage and its polarity. Resonances with a high-quality factor (and thus a small full width half maximum $\delta {\omega }_{{{{{{{{\rm{res}}}}}}}}}=2\pi \times \delta {f}_{{{{{{{{\rm{res}}}}}}}}}$) are favorable as they minimize the so-called switching voltage *V*_eo_ = *V*_switch_ that is defined as the voltage that is necessary to fully shift the resonance away from its unbiased value, which occurs when $\delta {\omega }_{{{{{{{{\rm{res}}}}}}}}}={{\Delta }}{\omega }_{{{{{{{{\rm{eo}}}}}}}}}$. In conditions of high-Q, an optical beam experiences full modulation of its intensity or phase even for low *V*_switch_. The frequency shift can be derived from the phase modulation Δ*ϕ*_eo_ = Δ*ω*_eo_*t*_int_ introduced by the Pockels effect, where ${t}_{{{{{{{{\rm{int}}}}}}}}}=\frac{2\pi }{{\gamma }_{{{{{{{{\rm{rad}}}}}}}}}}=\frac{2\pi }{\delta {\omega }_{{{{{{{{\rm{res}}}}}}}}}}$ is the interaction time of the optical beam with the control field within the nonlinear material and *γ*_rad_ the radiative loss rate of the optical field out of the interaction region, into the far-field. Recent breakthroughs in the engineering of high-Q plasmonic resonators^39^ or Mie resonators^40^ from silicon nanoantennas^41^ or bound states and quasi-bound states in the continuum (quasi-BICs)^42^ have showcased compelling free-space candidates that now routinely reach quality factors on the order of few hundreds to few thousands. They were applied across the entire spectrum, from the visible to the terahertz^33^, in both metasurface and waveguide geometries^43^. Here, we harness the unique properties of such quasi-BICs for hybrid silicon-organic electro-optic modulators that feature a small footprint and low dimensions (as illustrated in Fig. 1a) and that preserve a Q factor up to 550 even when homogeneously integrated with high-performance electro-optic molecules and interdigitated driving electrodes. A highly efficient electro-optic transduction is made possible by state-of-the-art *χ*^(2)^ organic molecules JRD1 in polymethylmethacrylate (PMMA)^44^ that have a low absorption in the near-infrared and are spatially located within the high-field areas of the optical nearfield. By judicious three-dimensional engineering, we incorporate metallic coplanar waveguides (CPW) that provide GHz-speed driving fields, without compromising the losses significantly and thereby lowering the performance. In short, the physics behind quasi-BICs relies on confined modes that can exist inside a continuum. They were shown to appear in structures of the kind shown in Fig. 1a and their narrow linewidth originates from the low coupling of the electric dipole fields in the nearfield of the resonators with propagating modes. Our geometry (discussed in detail in ref. ^42^) explores symmetry breaking as a mean to influence the linewidth of the optical modes. Finally, we benchmark the performance of quasi-BIC modes for free-space transduction against guided-mode resonances (GMRs) that can arise in similar nanostructures. GMRs appear due to the scattering of incident light by the silicon pillars that form a grating into orders whose scattering angle coincides with the direction of the propagation vector of guided modes. In this case, propagating modes in the slab formed by JRD1:PMMA are excited efficiently by the grating.

## Results

**Fig. 2:**
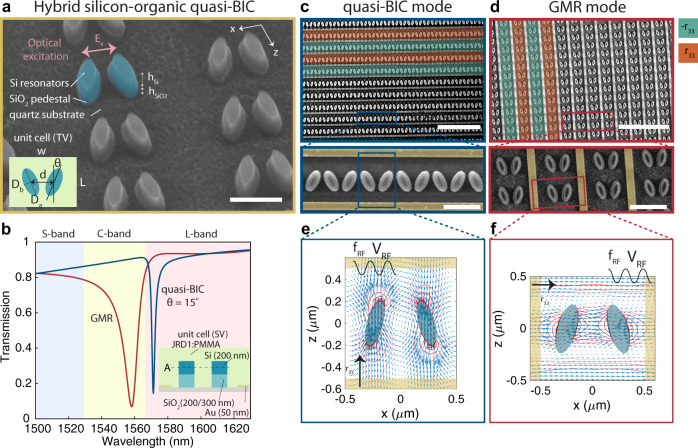
Hybrid silicon-organic free-space electro-optic modulators based on Mie resonances for C- and L-bands. **a** A single electro-optic modulator is made from a rectangular array of silicon nanoresonators patterned onto a quartz substrate on top of a silicon dioxide pedestal, here shown prior to the deposition of the metallic electrodes and the organic electro-optic layer (green) which is applied post-fabrication by spin-coating and covers the nanoresonators. Inset shows the top view (TV) of one single unit cell. Scale bar = 500 nm. This geometry can sustain two distinct types of resonances, quasi-bound states in the continuum (quasi-BICs) and guided-mode resonances (GMR), shown in **b**, with corresponding geometries as in **c**, **d**. Inset shows the side view (SV) of one unit cell. The two types of resonances are excited by an incident beam that is *x*-polarized and have distinct distributions of the near-fields of the resonators, shown in **e**, **f** (cross-section A of SV). While the quasi-BIC mode is circulating in the nearfield and has a dominant component along the *z*-axis (hence perpendicular to the excitation polarization), the guided-mode resonance is predominantly *x*-polarized (as the excitation). Given this vectorial orientation of the optical fields in the nearfield of the resonators, metallic electrodes are deposited in between each row of ellipses and are oriented along the *x*-axis for the quasi-BICs and along the *z*-axis for the GMR, as shown in **b**, **c** (scale bars upper picture = 5 μm and lower pictures = 1 μm). The interdigitated electrodes serve for the activation of the JRD1:PMMA layer by electric field poling and for the application of DC and RF tuning fields. Black arrows indicate poling direction.

An array of elliptical silicon resonators is patterned on a quartz substrate and gold interdigitated electrodes are deposited around the resonators and then covered by the high-quality active organic layer. The embedded array has a sub-wavelength thickness and operates in a transmission geometry where an optical field is normally incident from free space. Fabricated devices are shown in Fig. 2 and the fabrication protocol is discussed in the methods and sketched in Supplementary Fig. S1. The scanning electron micrographs (SEM) of Fig. 2a, c, d show the array of silicon resonators prior to and after the deposition of the metallic electrodes. The layer of organic electro-optic molecules consists of JRD1:PMMA of 50%wt, has a lower refractive index than silicon ($\tilde{n}=n+ik$, with *n* = 1.67 and *k* = 5 × 10^−5^ at ${\lambda }_{{{{{{{{\rm{res}}}}}}}}}=1550\,{{{{{\rm{nm}}}}}}$), and is not shown here. Wavelength-, voltage-, and concentration-dependent properties of the active layer are extensively reported in ref. ^24^ and its associated supplementary information. By choice of the exact geometrical parameters of one unit cell of the array illustrated in the insets of Fig. 2a (TV = top view) and Fig. 2b (SV = side view), the silicon nanostructures can be engineered to exhibit quasi-BIC and GMRs in the C- and L-telecom bands, as shown in Fig. 2b. While their linewidth is clearly very distinct, both types of optical modes are localized mostly in the layer of organic molecules and outside the high-index silicon material, as demonstrated by their simulated field profiles in Fig. 2e, f, where the arrows denote the orientation of the electric field in the plane A of the resonators. The simulations are done with CST Microwave Studio using the frequency domain solver and periodic boundary conditions. Moreover, we chose to pattern the silicon resonators of height *h*_*Si*_ = 200 nm on top of an elliptical pedestal from silicon dioxide of height ${h}_{Si{O}_{2}}=$ 200 or 300 nm, as visible from both the SEM figures and the cross-section of one unit cell shown in Fig. 2b. This step is essential to minimize the overlap and thereby the losses of the optical field with the metallic electrodes. Simulated cross-sections of the optical field are displayed in Supplementary Fig. S3 to demonstrate its localization in the nearfield of the silicon resonators.

We note a particular feature of the two modes considered here. While both resonances are excited with *x*-polarized light, the optical nearfield of the resonators is mainly *z*-polarized for the quasi-BIC mode and *x*-polarized for the GMR mode. This fact explains our choice of electrode orientation that is different for the two resonances: for the quasi-BIC structure, the electrodes are parallel to the *x*-axis, while for the GMR structure, they are parallel to the *z*-axis. This orientation maximizes the alignment of the optical nearfield parallel to the applied RF-field, oriented perpendicularly to the electrodes, and allows us to exploit the *r*_33_ coefficient of the electro-optic tensor of the JRD1:PMMA layer. We note that in the case of the organic layer used here, the orientation of the electro-optic tensor with respect to the geometrical coordinate system of the sample is established post-fabrication, by electric field poling, a procedure during which the molecules orient along a DC electric field^13,24,45^ that is applied via the gold electrodes. By definition, in the organic layer utilized here, *r*_33_ corresponds to the direction of the poling field. We provide in Supplementary Fig. S3 electrostatic simulations of the poling fields for the two geometries. The orientation of the poling fields and their relative strength is indicative of the orientation and level of alignment of the organic electro-optic molecules. Since in our case, the entire array is poled at once by interdigitated electrodes, the electro-optic coefficient *r*_33_ alternates in sign from one electrode period to the next as illustrated by the green and red areas in Fig. 2c, d yielding an overall in-plane periodically poled JRD1:PMMA film with a typical *r*_33_ = 100 pm/V as was previously demonstrated and characterized in detail in ref. ^24^. As a result, this particular structure allows us to maximize the overlap factor Γ_*c*_ for both modes.

**Fig. 3:**
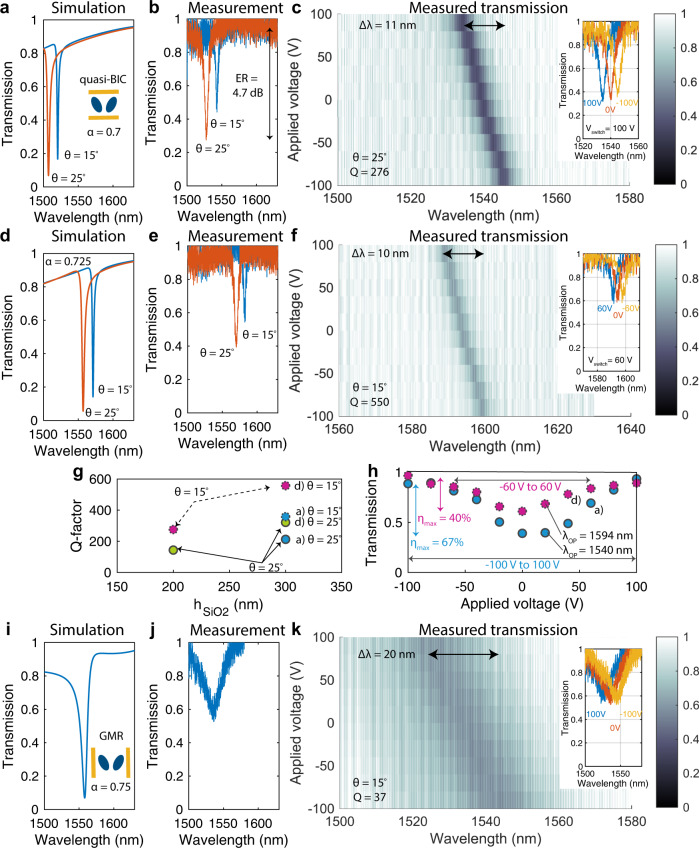
DC tuning properties of Mie-based modulators. **a**, **b** and **d**, **e** Experimental transmission results are compared to simulated transmission curves for various geometries of electro-optic modulators based on quasi-BIC structures. We find, as expected, by experiment and simulation, that the geometrical scaling factor *α* shifts the resonances within the telecom band. In addition, the asymmetry angle *θ* influences the linewidth of the resonances. **c**, **f** are DC tuning maps of the electro-optic modulators for (*α*, *θ*) = (0.7, 25°) and (*α*, *θ*) = (0.725, 15°), respectively. Insets show three distinct curves at 0 V, and *V*_switch_ = ± 100 V and ± 60 V, respectively. **g** Experimentally extracted quality factors for two distinct heights of the silicon dioxide pedestal are compared and we find that an increase in height from 200 to 300 nm leads to an increase in the quality factor. Dashed circles represent quasi-BIC structures with *θ* = 15° and full contour circles represent *θ* = 25°. The circles for *h*_*SiO*2_ = 300 nm denote the measurements as labeled to the right of the circles. The circles for *h*_*SiO*2_ = 200 nm represent measurements of equivalent structures with *h*_*SiO*2_ = 200 nm. **h** Detailed voltage-dependent transmission curves are reported for two exemplary operating wavelengths for the case of the two device geometries discussed in **a**, **d**. Full switching of transmission is achieved for both geometries. **i**, **j** In contrast, GMRs in the same structure have much broader linewidths, demonstrated by experiment and simulation. **k** Their resonance wavelength can be tuned over ${{\Delta }}{\lambda }_{{{{{{{{\rm{res}}}}}}}}}=20$nm. Q factors and asymmetry angle *θ* are indicated for all colormaps. ER extinction ratio.

In the following, we first present experimental tuning properties of the hybrid silicon-organic free-space modulators when a DC voltage *V*_eo_ is applied to the interdigitated electrodes uniformly across the entire array (*f*_RF_ = 0). In Fig. 3a–f we report the experimental results for operation on a quasi-BIC mode engineered in the C- or L-telecom band with geometrical dimensions as defined in the inset of Fig. 2a. These dimensions depend on a geometrical scaling parameter *α* as follows: *W* = 1.32 × *α* μm, *L* = 1.4 μm, *D*_*a*_ = 2 × 0.33 × *α* μm, *D*_*b*_ = 2 × 0.11 × *α* μm, *d* = 0.66 × *α* μm, and provided in Methods. A particular feature of the quasi-BIC mode is that its Q factor is highly dependent on the asymmetry angle *θ*: in the absence of material losses, the quality factor increases toward infinity in the limit of *θ* = 0. We report in Supplementary Fig. S4 the simulated dependence of the transmission on *θ* for the fabricated structures. Noting that in the presence of losses, at high-Q factors the resonance depth also decreases (eventually leading to less intensity modulation), we choose *θ* = 15° and 25°. We confirm by experiment the expected increase of quality factor when reducing the angle from *θ* = 25° (*Q* = 212 and *Q* = 320 for *α* = 0.7 and *α* = 0.725, respectively) to *θ* = 15° (*Q* = 357 and *Q* = 557 for *α* = 0.7 and *α* = 0.725, respectively), see Fig. 3g. The measurements were performed on structures similar to Fig. 2c prior to the electric field poling of the devices. Furthermore, the measured redshift of the resonance with decreasing *θ* is well reproduced by our simulations. In Fig. 3c we report the DC tuning characteristics of the modulator based on quasi-BIC modes as a function of applied voltage *V*_eo_ of the structure with *α* = 0.7 and *θ* = 25°, after the non-linearity of the electro-optic molecules is established by the electric field poling. First, we observe at *V*_eo_ = 0 V a shift of the resonant wavelength by 12 nm in the poled sample (${\lambda }_{{{{{{{{\rm{res}}}}}}}}}=1540$ nm) compared to the unpoled sample (${\lambda }_{{{{{{{{\rm{res}}}}}}}}}=1528$ nm). The experimental Q factor of the poled sample is *Q* = 276. Second, we find that under an applied voltage change from *V*_eo_ = 100 V to *V*_eo_ = −100 V, the resonance shifts linearly with applied voltage, as expected (according to $\frac{{{\Delta }}{\lambda }_{{{{{{{{\rm{res}}}}}}}}}}{{\lambda }_{{{{{{{{\rm{res}}}}}}}}}}=-\frac{1}{2}{n}_{{{{{{{{\rm{mat}}}}}}}}}^{2}{r}_{33}E{{{\Gamma }}}_{c}$, with $E=\frac{{V}_{{{{{{{{\rm{eo}}}}}}}}}}{L}$) up to a maximum of ${{\Delta }}{\lambda }_{\max }=11$ nm, which suffices to satisfy ${{\Delta }}{\omega }_{{{{{{{{\rm{eo,100V}}}}}}}}}-{{\Delta }}{\omega }_{{{{{{{{\rm{eo,-100V}}}}}}}}} \sim 2\times \delta {\omega }_{{{{{{{{\rm{res}}}}}}}}}\ge \delta {\omega }_{{{{{{{{\rm{res}}}}}}}}}$ (see inset). We introduce the switching voltage *V*_switch_ as a figure of merit that quantifies the voltage that is necessary to switch the transmission between its maximal and its minimal value (which corresponds conceptually to the widely used *V*_*π*_). We find a *V*_switch_ = 100 V to be sufficient to tune the absolute intensity transmitted through the sample at a chosen operation wavelength *λ*_*OP*_ between its minimum at ${T}_{\min }=30 \%$ and its maximum at ${T}_{\max }=90 \%$ of its maximum value (shown also in Fig. 3h). This corresponds to a maximal modulation depth ${\eta }_{\max }=\frac{{{\Delta }}T}{{T}_{\max }}=67 \%$, where ${{\Delta }}T={T}_{\max }-{T}_{\min }$ is the total modulation change. This corresponds to an extinction ratio (ER) of 4.7 dB. In a second example shown in Fig. 3f, we choose to operate on the narrower resonance present when *θ* = 15° and *α* = 0.725. In this case, we report a maximal tuning of the resonance by ${{\Delta }}{\lambda }_{\max }=10$ nm, which corresponds to a ${{\Delta }}{\omega }_{eo,100V}-{{\Delta }}{\omega }_{eo,-100V} \sim 3.46\times \delta {\omega }_{{{{{{{{\rm{res}}}}}}}}}\ge \delta {\omega }_{{{{{{{{\rm{res}}}}}}}}}$. Also in this case, at *V*_eo_ = 0 V we observe a shift of the resonant wavelength by 11.6 nm of the poled sample (${\lambda }_{{{{{{{{\rm{res}}}}}}}}}=1594$ nm) compared to the unpoled sample (${\lambda }_{{{{{{{{\rm{res}}}}}}}}}=1582.4$ nm). The Q factor of the poled sample is *Q* = 550. Importantly, in this case, because of the higher Q factor, a voltage change of only *V*_eo_ = 60 V or *V*_eo_ = −60 V suffices to fully tune through the entire resonance (see inset). Consequently, we find that a voltage *V*_switch_ = 60 V is sufficient to switch the absolute intensity transmitted through the sample between its minimum at 60% and its maximum at 100% of its maximum value. In this case, ${\eta }_{\max }=40 \%$, see Fig. 3h.

To contrast these two examples, we now analyze in Fig. 3i–k the DC tuning behavior when we operate the elliptical resonators on the GMR modes introduced in Fig. 2d, f with geometrical dimensions (*W* = 1.4 μm, *L* = 1.32 × *α* μm, *D*_*a*_ = 2 × 0.33 × *α* μm, *D*_*b*_ = 2 × 0.11 × *α* μm, *d* = 0.66 × *α* μm) as provided in the Methods. We find from both experiments and simulations a much broader resonance with an experimental *Q* = 37, where a voltage change of *V*_eo_ = 100 V to *V*_eo_ = −100 V tunes the resonant wavelength over a maximal range of ${{\Delta }}{\lambda }_{\max }=20$ nm. This value is approximately twice larger than what we find for the quasi-BIC modes, and can be attributed to a more efficient interaction enabled by the *r*_33_ electro-optic coefficient due to higher alignment of the nearfield of the nanoresonators with the tuning field (see side-by-side mode profiles and poling/tuning field simulations in Supplementary Fig. S3). However, the broad linewidth of the resonance would require a *V*_switch_ larger than 100 V, thereby demonstrating that GMR can be utilized in scenarios where a large tuning of broad resonances is preferred over a large intensity modulation, as may be the case of modulating broadband emission. Notably, the achieved tuning is approximately twice as larger as previous reports that investigated GMR inside a single organic layer from JRD1:PMMA^24^, thereby underlining the relevance of sub-wavelength resonators for high-performance free-space modulators.

**Fig. 4:**
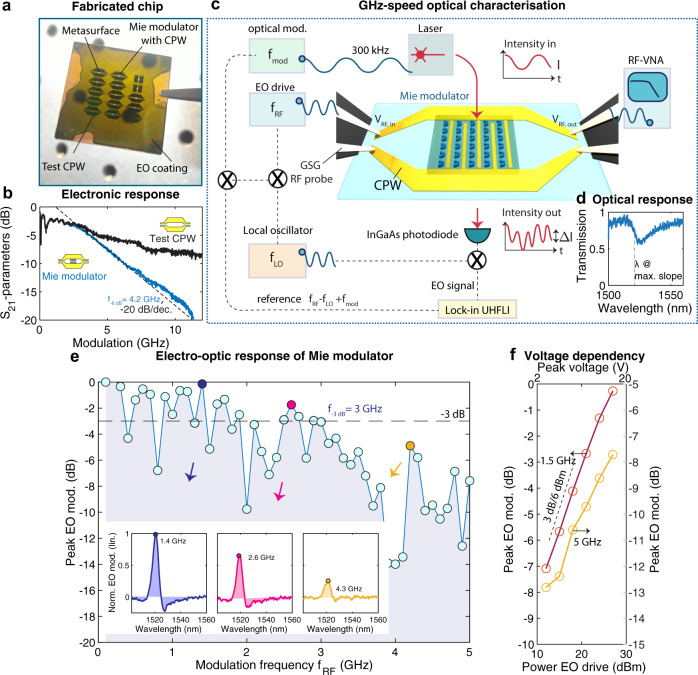
GHz-speed properties of the Mie modulators. **a** Picture of a fabricated chip shows Mie modulators that are integrated with GHz coplanar waveguides (CPW). Also visible are test CPW. **b** Electronic scattering parameters *S*_21_ of Mie modulators are compared to test CPW. The *S*_21_ are measured using a vector network analyzer (VNA) connected to the sample by high-frequency cables and high-speed microwave GSG (ground-source-ground) probes (one ground floating) and exhibits a cut-off of *f*_−6*dB*_ = 4.2 GHz owing to the intrinsic RC bandwidth. RF cable losses are deducted from the *S*_21_ response. Beyond 4.2 GHz, only the Mie modulators exhibit a decay of –20 dB/decade (much less steep roll-over for the test CPW). **c** Optoelectronic experimental setup. The electronic characteristics are measured in a transmission configuration using the VNA, and the wavelength-resolved electro-optical (EO) modulation is measured using a lock-in amplifier. A double modulation scheme combined with a local oscillator (LO) is used, where the laser emission is modulated at the source and the Mie modulators (details in the methods) are actuated electronically by the RF source. **d** Resonance of sample (*h*_*SiO*2_ = 200 nm, *θ* = 25°). **e** Peak electro-optic modulation amplitude for frequencies *f*_RF_ up to 5 GHz. We find a 3-dB electro-optic bandwidth of *f*_*EO*,−3*dB*_ = 3 GHz. Insets: wavelength-resolved modulation strength for several values of *f*_RF_, the peak values have been utilized to plot the data in **e**. **f** Peak electro-optic modulation amplitude for different modulation voltages (reported as power in dBm), at 1.5 and 5 GHz, the latter well beyond the electro-optic bandwidth.

Finally, we analyze the GHz-speed properties of the Mie modulators based on quasi-BICs (*α* = 0.675 and *h*_*SiO*2_ = 200 nm) in Fig. 4. A photograph of several fabricated devices is provided in Fig. 4a and displays two sets of devices: Mie modulators fitted with interdigitated tuning electrodes that are connected to GHz-speed CPW and test devices that consist only of the CPW (no metasurface and no interdigitated electrodes). We first characterize these two structures electrically using a vector network analyzer (VNA) that outputs the scattering matrix, including the amount of transmitted RF power, characterized by *S*_21,*dB*_, using the setup shown in Fig. 4c. We find a −6 dB cut-off of the Mie modulators at *f*_−6*dB*_ = 4.2 GHz, after which a roll-over of −20 dB/decade is observed, which agrees well with the RC time constant of the interdigitated electrode array of the Mie modulators (see Methods). After the roll-over, the voltage across the modulators drops toward zero. This is in contrast to the test CPW that does not feature such decay. RF cable losses are deducted from the *S*_21,*dB*_ response. Then, we characterize the GHz-speed electro-optic tuning properties of the Mie modulators around their resonance with optical transmission characteristics as shown in Fig. 4d. We apply a drive field ${V}_{{{{{{{{\rm{RF}}}}}}}}}={V}_{{{{{{{{\rm{eo}}}}}}}}}\times \sin (2\pi {f}_{{{{{{{{\rm{RF}}}}}}}}}t)$. We use a double modulation scheme in combination with a local oscillator and lock-in detection to characterize the sample up to 5 GHz, above the lock-in bandwidth. Details of the experimental setup are given in the Methods and photographs of the lab setting in Supplementary Fig. S2. In Fig. 4e, we first report the peak electro-optic modulation *η*_*peak*,*dB*_ as a function of frequency *f*_RF_ (we note that here the peak modulation amplitude has been normalized to its value at 100 MHz *η*_peak_(*f*_RF_ = 100 MHz) and computed using ${\eta }_{peak,dB}=10\;{\log }_{10}\frac{{\eta }_{{{{{{{{\rm{peak}}}}}}}}}({f}_{{{{{{{{\rm{RF}}}}}}}}})}{{\eta }_{{{{{{{{\rm{peak}}}}}}}}}(f_{{{{{{{\rm{RF}}}}}}}}=100\,MHz)}$). We find that the sample electro-optic bandwidth is *f*_*EO*,−3*dB*_ = 3 GHz, and that at *f*_RF_ = 5 GHz, the modulation amplitude is approximately 7.75 dB lower than the maximum. The discrepancy between the electronic bandwidth and the electro-optic bandwidth can be ascribed to attenuation in the cable from the photodiode to the lock-in amplifier, passing several stages of mixers, which was not accounted for in this experiment. The oscillations in the electro-optic response may originate from electronic resonances within these cables. We only accounted for attenuation from the RF source to the chip. In the inset of Fig. 4e, we show wavelength-resolved EO modulation for three distinct modulation frequencies (marked by the colored dots, *f*_RF_ = 1.4, 2.5, and 4.3 GHz at an RF power of 27 dBm at the source). For each wavelength, we normalized the absolute electro-optic modulation to the transmission through the unbiased sample. We find, as expected, that the modulation strength peaks on one side of the asymmetric resonance, more specifically at the wavelength *λ* with the highest slope in the transmission and that it changes sign at the resonance wavelength ${\lambda }_{{{{{{{{\rm{res}}}}}}}}}$. Moreover, we note that a modulation can be measured beyond the 3-dB cut-off, e.g., at *f*_RF_ = 4.3 GHz. In Fig. 4f we investigate the dependence of the modulation amplitude on the drive power at frequencies 1.5 and 5 GHz and observe an approximately linear behavior as expected.

## Discussion

Our work is the first step toward a class of free-space electro-optic modulators that provide compact and efficient intensity modulation of free-space beams at telecom frequencies. They leverage the unique design flexibility of high-Q sub-wavelength resonators covered by an active electro-optic layer to achieve efficient GHz-speed tuning by incorporation with microwave-compatible electrodes. In the future, the fabrication procedure we propose may allow exploration of sub-wavelength resonators not only of other geometries (e.g., to achieve phase-only modulation^46^ or polarization modulation) but also of other materials commonly used for metasurfaces such as oxides (e.g., titanium dioxide or hafnium dioxide), metals or semiconductors (e.g., germanium). The active organic layer is then applied post-fabrication. Also at DC voltages, the use of quasi-BIC modes allows us to achieve a tuning over 31% of the C-telecom band, corresponding to 11 nm, while GMRs achieve a tuning up to 20 nm. Prior work that concentrated on evaluating various properties of the organic layer for free-space active photonics^24^ was limited to using the electrode array also for the purpose of introducing the optical resonance, unlike in our case where the optical properties can be engineered by the geometry of the silicon resonators independently of the electrode array that serves solely the purpose of applying the tuning microwave fields. This advancement leads to tuning the GMRs over a twice larger wavelength range when using sub-wavelength resonators, due to improved overlap of the optical mode with the active organic layer. Furthermore, the interdigitated electrode design features low capacitance and hence allows operation at microwave speeds, with the current cut-off frequency being linked to the total area of the device alone. This demonstrated switching speed could promote further research of direct integration with high-frequency electronics, e.g., for time-dependent and high-speed on-demand control of light, for example for vortex beam generation^47,48^ or for time-resolved microscopy and sensing^49^. In this respect, while our demonstration clearly demonstrates that driving voltages up to 100 V can be applied to the structure, in the future, it may become desirable to operate ultrathin free-space modulators at CMOS voltages with higher ER. In Supplementary Information S5, we outline possible routes that may be taken towards this goal, notably that both higher ERs or a reduction of the switching voltage to 12 V may come into reach by replacing the electrode material with transparent conductive oxides, as discussed in Section S3.D. Furthermore, in the Supplementary Information of this work, we provide the first proof of principle spatial light modulator realized from quasi-BICs as a mean to achieve spatial multiplexing. As a result of these cumulative advances, our design already exhibits superior key figures of merit over other reported electro-optic metasurface platforms, as outlined in Supplementary Information S5. If even higher modulation speeds are desired in the future, the intrinsic RC time constant of the device could be reduced by a factor of 10 and would potentially allow operation up to 30 GHz by reducing the in-plane footprint of the devices from a current approximative area of 330 × 330 μm^2^ to potentially 100 × 100 μm^2^. Finally, the achieved relative bandwidth tuning of 0.7% in combination with the high-Q factor may allow us to investigate additional emergent nonlinear optical phenomena. Their high-speed characteristics may become useful in the area of time-varying^50^ and spatio-temporal^5,51^ metasurfaces with electro-optic materials^51^, and provide an alternative path to magnet-free isolators beyond optomechanical^52^ or piezoelectric^53^ actuation. Alternatively, the device architecture may benefit, e.g., second harmonic generation that also requires highly confined fields in combination with highly performant nonlinear materials^54,55^, which has so far not been demonstrated in hybrid silicon-organic systems of this kind.

## Methods

### Nanofabrication of the hybrid silicon-organic electro-optic modulators

The structures discussed in this study use in part standard nanofabrication technique used for the silicon-on-insulator platform. These are complemented by a final step in which the organic active layer is applied to the structure and subsequently activated by electric field poling. The fabrication flowchart is shown in Supplementary Fig. S1. In short, a multilayer of amorphous silicon (of thickness 200 nm) on silicon dioxide (of thickness 200/300 nm) is deposited by chemical vapor deposition onto a quartz substrate. Then, elliptical nanostructures are patterned by electron beam (e-beam, Elionix 125, 1 μA current) lithography onto ZEP 520A resist (spincoated at 3000 rpm) and act as a etch mask in a subsequent two-step fluoride-based reactive ion etching (SF_6_, C_4_F_8_), during which the silicon is first etched and then the silicon dioxide is etched using the same resist mask. After etching, the gold electrodes (15 nm titanium, 35 nm gold) are deposited via e-beam evaporation and subsequent lift-off of ZEP 520A resist in overnight remover PG at 80 °C. Finally, a mixture of 50%wt JRD1:PMMA (where PMMA = polymethylmetacrylate) dissolved in 5%wt 1,1,2-trichlorethane is deposited by spin coating at 1000 rpm to the structure to reach a layer thickness of 600–700 nm. After coating, the organic film is dried in an under-vacuum oven at 80 °C for 24 h. After drying, the organic film is rendered electro-optically active by electric field poling, a procedure explained, e.g., here^13^ during which the sample is heated up above the glass temperature of the organic layer (95 °C) and quickly cooled down under an applied poling field on the order of *E*_*pol*_ = 100 V/μm. During this procedure, the electro-optic molecules JRD1 undergo a reorientation from random (after spin-coating) to being aligned with the poling field lines (that have a three-dimensional characteristic discussed in greater detail below and in Supplementary Fig. S3), owing to their hyperpolarisability. The wavelength-dependent electro-optic properties, the wavelength-dependent and concentration-dependent refractive indices of the JRD1:PMMA mixture are discussed in great detail and provided in the Supplementary Information of ref. ^24^.

### Optical and electronic properties of the electro-optic modulators

The performance of the modulators we demonstrate arises primarily from the fact that the structures we propose support high-Q modes that have a high overlap with the active nonlinear material. We compute the quality factor using the formula $Q=\frac{{\omega }_{{{{{{{{\rm{res}}}}}}}}}}{\gamma }$ with *γ* the total loss rate of the resonance which we extract by fitting a Lorentzian lineshape to the transmitted power of the shape $I(\omega )=\frac{A}{{(\omega -{\omega }_{{{{{{{{\rm{res}}}}}}}}})}^{2}+{(\frac{\gamma }{2})}^{2}}+B$, where A, B, ${\omega }_{{{{{{{{\rm{res}}}}}}}}}$ and *γ* are fitting parameters. In Supplementary Fig. S3, we report the simulated distribution of both the optical fields as well as the poling fields (which generate the *χ*^(2)^ non-linearity in the organic coating of our hybrid structures). The latter distribution also corresponds closely to the driving DC and RF fields that are applied to the device under test to trigger the electro-optic effect in the modulators. All simulations are done with CST Microwave Studio: the optical simulations are performed using the frequency domain solver, while the DC simulations used the electrostatic solver. The detailed dielectric constant of the organic layer can be found for DC and optical frequencies in ref. ^24^.

### Geometrical properties of silicon nanostructures

We chose the following dimensions for the quasi-BIC structures: *W* = 1.32 × *α* μm, *L* = 1.4 μm, *D*_*a*_ = 2 × 0.33 × *α* μm, *D*_*b*_ = 2 × 0.11 × *α* μm, *d* = 0.66 × *α* μm, *θ* = 15°, 25°, *α* = 0.7 (Fig. 3a–c) and *α* = 0.725 (Fig. 3d–f) and number of periods *N*_*x*_ = 360 and *N*_*z*_ = 240 along the *x*- and *z*-axis, respectively. In all cases, the gold electrodes have a width of 200 nm, a length of 330 μm and a height of 50 nm (15 nm titanium and 35 nm gold). We chose the following dimensions for the GMR structures: *W* = 1.4 μm, *L* = 1.32 × *α* μm, *D*_*a*_ = 2 × 0.33 × *α* μm, *D*_*b*_ = 2 × 0.11 × *α* μm, *d* = 0.66 × *α* μm, *θ* = 15°, number of periods *N*_*x*_ = 240 and *N*_*z*_ = 360 and *α* = 0.75. Also, *h*_*SiO*2_ = 200 nm. For the high-frequency measurements shown in Fig. 4, we used a quasi-BIC structure with *α* = 0.675.

### High-frequency optoelectronic characterization of Mie modulators

First, we characterize the electronic ${S}_{21,dB}=20\;{\log }_{10}\frac{{V}_{RF,out}}{{V}_{RF,in}}$ parameters using a VNA (Agilent E8364B) by contacting two GSG probes from GGB (Picoprobe 40A series, DC to 40 GHz) to the CPW of the modulators. Second, a double modulation scheme (*f*_*mod*_, *f*_eo_) in combination with a local oscillator (LO, *f*_*LO*_) is used to characterize the high-frequency electro-optic tuning properties of the Mie modulators. The laser is internally modulated at *f*_*mod*_ = 300 kHz, 50% duty cycle, and full intensity modulation using an external source (pulser Agilent B114A). The electro-optic modulators are modulated at the speeds reported in Fig. 4 using a second RF source that outputs a sinusoidal signal (Hittite Microwave Corporation HMC-T2100B, 10 MHz to 20 GHz). The modulated laser intensity is detected by a photodiode (Newport 1544-A, bandwidth 12 GHz). A third RF source (Wiltron Anritsu 68347B, 10 MHz to 20 GHz) is used as a local oscillator at frequency *f*_*LO*_ = *f*_RF_ + 41 MHz to mix down the photodiode signal to an intermediate frequency of *f*_*IF*_ = 41 MHz, irrespective of the modulating frequencies *f*_RF_ (mixer ZMF-2-S+, bandwidth 1–1000 MHz and mixer ZX05-C42-S+, bandwidth 1000–4200 MHz, both from Mini-circuits). The downmixed photodiode signal is recorded by a high-frequency lock-in amplifier (UHFLI from Zurich instruments, with a maximal demodulation frequency 600 MHz). The second set of mixers (mixer ZMF-2-S+ from Mini-circuits, bandwidth 1–1000 MHz and mixer ZLW-1-1+ from Mini-circuits, bandwidth 0.1–500 MHz) is used to mix the intermediate frequency with the modulation frequency to form the reference signal at *f*_*ref*_ = *f*_*IF*_ + *f*_*mod*_ that is used to demodulate the downmixed photodiode signal and to report the modulated intensity. This double modulation scheme combined with the local oscillator is necessary to unambiguously detect the electro-optic modulation of the sample in frequency ranges that are larger than the cut-off of the lock-in amplifier. The light incident onto the Mie modulators is collimated prior to the sample (diameter 6 mm) and then focused onto the sample using a lens with a focal length of 100 mm. The sample is placed in the focus of the beam and its position is adjusted using an xyz stage. A linear polarizer filters any polarization components that are not parallel to the *x*-axis.

### RC time constant of Mie modulators

The Mie modulators investigated in this work have dimensions as discussed above. Their switching speed is mainly determined by the capacitance of the interdigitated array of electrodes, loaded with the organic material and with silicon pillars, and by the 50 Ω resistance of the source. We consider a simplified model^56^ for the computation of the capacitance per period, per unit length, using the formula below:

1 $${C}_{per}/L=\frac{{\epsilon }_{0}({\epsilon }_{Si{O}_{2}}+{\epsilon }_{JRD1:PMMA})}{4}\frac{K(\sqrt{1-{k}^{2}})}{K(k)}$$

where ${\epsilon }_{Si{O}_{2}}=3.75$, *ϵ*_*JRD*1:*PMMA*_ = 5, $k=\cos (\frac{\pi {w}_{electrodes}}{2{w}_{gap}})$ and *w*_*electrodes*_ = 0.2 μm is the width of the interdigitated electrodes, and *w*_*gap*_ = 1.2 μm, the gap between two electrodes. $K(k)=\int\nolimits_{0}^{1}\frac{dt}{{[(1-{t}^{2})(1-{k}^{2}{t}^{2})]}^{0.5}}$ are the elliptical integrals of the first kind. At a total length of *L* = 300 μm and a total number of periods of *N*_*z*_ = 240, the total capacitance is equal to *C*_*tot*_ = *N*_*z*_ × *C*_*per*_/*L* × *L* = 0.27 pF. With this, the RC cut-off frequency of the device is estimated analytically by assuming *R* = *R*_*source*_ + *R*_*device*_ (*R*_*source*_ = 50 Ω and *R*_*device*_ = 24 Ω the serial resistance of the device, measured on samples where all interdigitated electrodes were shorted) to *f*_−3*dB*,*calc*_ = 2.6 GHz and *f*_−6*dB*,*calc*_ = 4.5 GHz. We note however that there is a variance in the device resistance owing to the quality of the gold wires which might affect in turn the cut-off frequency. This formula does not consider the elliptical silicon resonators, which increase the capacitance compared to this estimated value and hence lower the RC cut-off frequency.

## Supplementary information

Supplementary information

## Data Availability

The main dataset generated in this study has been deposited in the Zenodo database under accession code 10.5281/zenodo.6458285.

## Code availability

The main code generated in this study has been deposited in the Zenodo database under accession code 10.5281/zenodo.6458285.
