# Supplementary material for: Gigahertz free-space electro-optic modulators based on Mie resonances
Source: Nat Commun. 2022 Jun 6;13:3170. doi: 10.1038/s41467-022-30451-z (PMC9170732; doi:10.1038/s41467-022-30451-z)
Supplement: Supplementary file 1 — Supplementary information [file 41467_2022_30451_MOESM1_ESM.pdf]

Supplementary information: Gigahertz free-space electro-optic modulators based on Mie resonances

Ileana-Cristina Benea-Chelmus

Harvard John A. Paulson School of Engineering and Applied Sciences, Harvard University, Cambridge, MA, USA and Hybrid Photonics Laboratory, École Polytechnique Fédérale de Lausanne (EPFL), Switzerland

Sydney Mason

Harvard College, Cambridge, MA, USA

Maryna L. Meretska

Harvard John A. Paulson School of Engineering and Applied Sciences, Harvard University, Cambridge, MA, USA

Delwin L. Elder

Department of Chemistry, University of Washington, Seattle, WA, USA

Dmitry Kazakov

Harvard John A. Paulson School of Engineering and Applied Sciences, Harvard University, Cambridge, MA, USA

Amirhassan Shams-Ansari

Harvard John A. Paulson School of Engineering and Applied Sciences, Harvard University, Cambridge, MA, USA

Larry R. Dalton

Department of Chemistry, University of Washington, Seattle, WA, USA

Federico Capasso

Harvard John A. Paulson School of Engineering and Applied Sciences, Harvard University, Cambridge, MA, USA

(Dated: April 13, 2022)

Abstract

Electro-optic modulators are essential for sensing, metrology and telecommunications. Most target fiber applications. Instead, metasurface-based architectures that modulate free-space light at gigahertz (GHz) speeds can boost flat optics technology by microwave electronics for active optics, diffractive computing or optoelectronic control. Current realizations are bulky or have low modulation efficiencies. Here, we demonstrate a hybrid silicon-organic metasurface platform that leverages Mie resonances for efficient electro-optic modulation at GHz speeds. We exploit quasi bound states in the continuum (BIC) that provide narrow linewidth ($Q = 550$ at $\lambda_{\mathrm{res}} = 1594$ nm), light confinement to the non-linear material, tunability by design and voltage and GHz-speed electrodes. Key to the achieved modulation of $\frac{\Delta T}{T_{\mathrm{max}}} = 67\%$ are molecules with $r_{33} = 100$ pm/V and optical field optimization for low-loss. We demonstrate DC tuning of the resonant frequency of quasi-BIC by $\Delta\lambda_{\mathrm{res}}$ =11 nm, surpassing its linewidth, and modulation up to 5 GHz ($f_{EO,-3\ dB} =$ 3 GHz). Guided mode resonances tune by $\Delta\lambda_{\mathrm{res}}$ =20 nm. Our hybrid platform may incorporate free-space nanostructures of any geometry or material, by application of the active layer post-fabrication.

S1. FABRICATION DETAILS

The structures discussed in this study use in part standard nanofabrication techniques used for the silicon-on-insulator platform and are complemented by a final step in which the organic active layer is applied to the structure and subsequently activated by electric field poling. The details are discussed in the Methods section of the main text, and a schematic of the fabrication protocol is provided in Fig. S1.

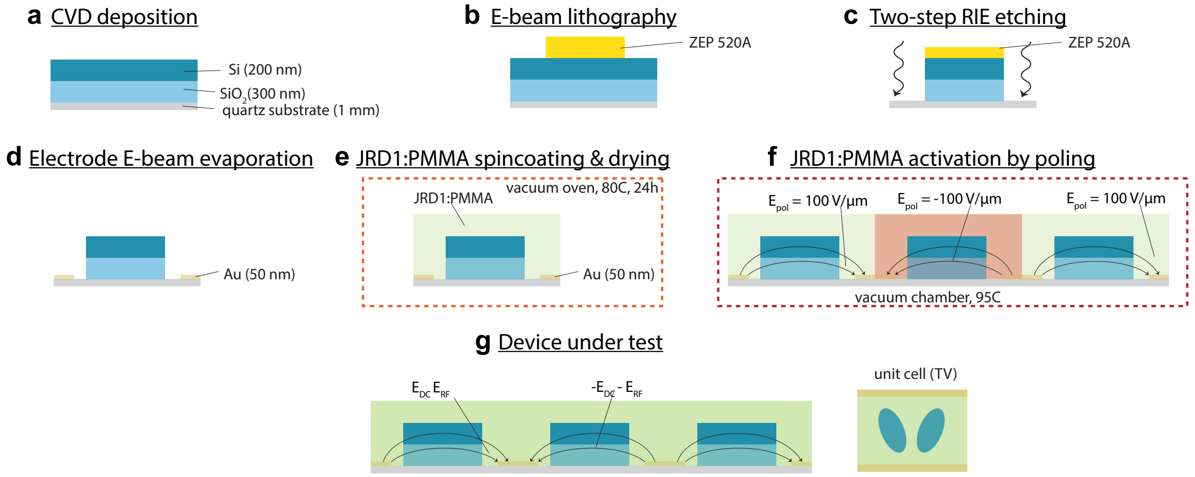

FIG. S1. **Fabrication protocol of hybrid silicon-organic modulators.** **a,** A multi-layer of amorphous silicon and silicon dioxide is deposited onto the substrate. **b,-c,** E-beam lithography is used to define the etch mask from cured ZEP 520A for the etching of the silicon and the silicon dioxide using RIE/ICP. **d,** Metallic contact electrodes are deposited via lift-off of ZEP 520A resist and subsequent electron beam deposition of a titanium/gold layer of 15/35 nm thickness. **e,** The active organic layer is applied to the nanostructures by spin coating and subsequently dried in a vacuum oven for 24 h at 80°. **f,** The organic layer is activated by electric field poling. Adjacent unit cells are poled in opposite direction, yielding an in-plane periodically poled electro-optic film, as previously introduced in Ref. [1]. **g,** While under applied driving electric field, the refractive index change is periodic across all unit cells, owing to the driving field that also switches sign from in two adjacent unit cells. CVD = chemical vapor deposition, RIE = reactive ion etching, Au = gold,

S2. HIGH FREQUENCY CHARACTERISATION SETUP

We built an electro-optical characterisation setup that is suitable to assess the performance of the samples both electrically and electro-optically under electrical driving fields that oscillate at microwave frequencies. In Fig. S2 we show camera pictures of the laboratory setup, featuring the characterization of the samples in transmission. A linear polarizer

and a lens with focal length $f = 100$ mm are used to create the linearly polarized incident optical beam (diameter 6 mm) and then further focus it at the plane of the Mie modulator. The path taken by the telecom laser light is shown by the red arrows. Microwave GSG probes inject the modulation fields via coplanar on-chip waveguides. They are positioned using xyz-RF probe stages. A fiber-coupled photodiode collects the transmitted light and the photocurrent signal is sent further to the set of mixers as described in the Methods in the main text. An InGaAs camera can be alternatively used to image and optimize the transmission of the telecom light through the modulator. Further technical details of the experimental setup and the data acquisition procedure are provided in the Methods.

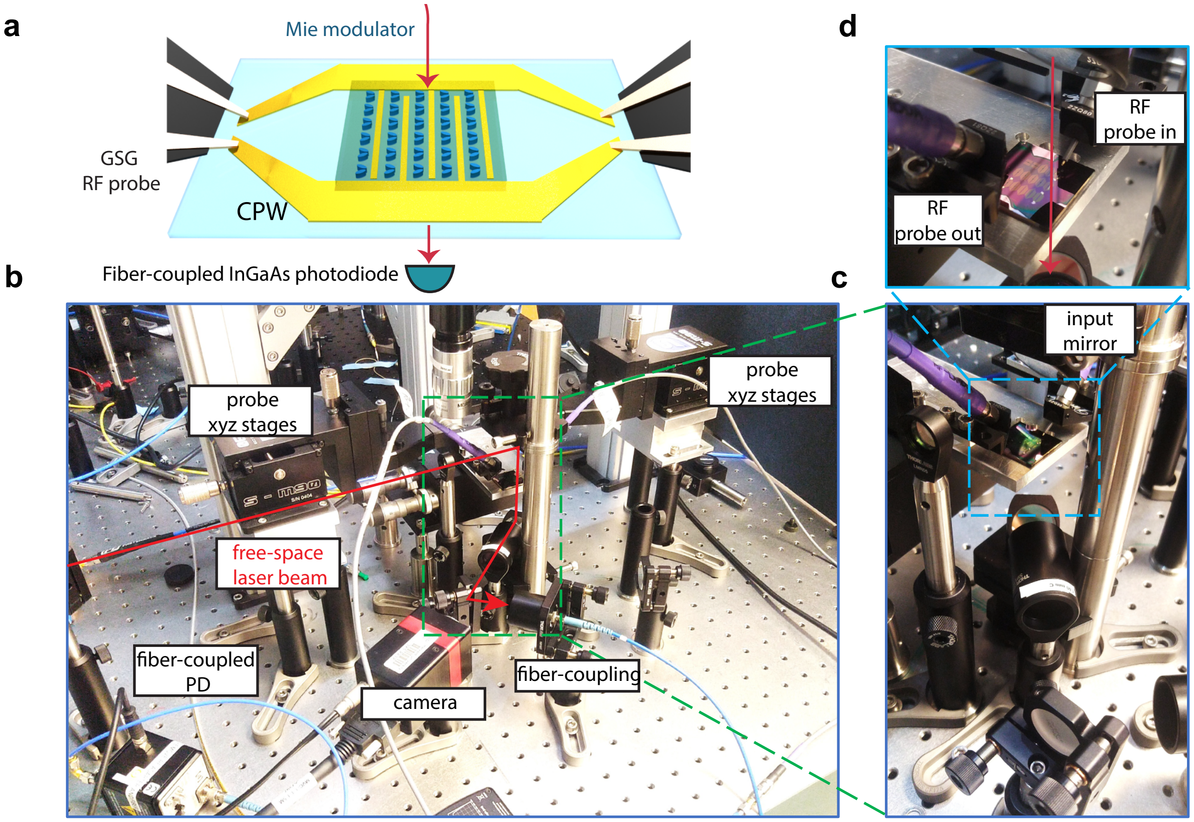

FIG. S2. **Pictures showing the GHz-speed setup used for electronic and electro-optic characterization of the free-space Mie modulators. a,** Simplified setup sketch. **b,-d,** Photograph of setup shows free-space coupling of telecom light (sketched by the red lines and arrows) to the metasurface that is placed horizontally, GSG probes connected to the devices as well as fiber-coupled high-speed InGaAs detector. PD = photodiode, CPW = coplanar waveguides, RF = radio frequency.

S3. PROPERTIES OF QUASI-BIC AND GMR RESONANCES

A. Optical and RF nearfields of the resonant Mie structures

The structures discussed in this work support quasi-bound states in the continuum and guided mode resonances. An efficient electro-optic transduction is achieved if the electro-optically induced frequency shift of the resonance is maximal, i.e. $\Delta\omega_{eo}(t) = -\frac{\Delta n(t)}{n_{mat}}\omega_{res}\Gamma_c = g_{eo}V_{RF}(t)$, with $g_{eo} = \frac{1}{2}n_{mat}^2 r_{33}\frac{1}{d}\frac{V}{}\omega_{res}\Gamma_c$. This requires that the strength and orientation of the optical field and the tuning DC/RF field are such that they interact strongly via the r_{33} component of the electro-optic tensor. This requires that the two fields have a strong overlap Γ_c with the nonlinear material in the nearfield of the resonators and that their vectorial orientation is as parallel as possible to the field lines of the DC fields. In Fig. S3 we show various crossections of the electric fields from electromagnetic and electrostatic simulations of the optical and DC fields. A maximal electro-optic transduction driven by r_{33} between the DC/RF tuning field and the optical field is ensured by the chosen electrode orientation. Due to the sub-wavelength nature of the structures we discuss, the vectorial distribution of RF modulating fields up to 5 GHz can be approximated by the electrostatic field distribution shown in Fig. S3.

We first investigate the field distribution at three distinct locations in the plane of the array of resonators: plane A is located 50 nm below the silicon resonators, plane B is located in the center of the silicon resonators and plane C is located 50 nm above the silicon resonators in the JRD1 layer. Furthermore, we provide also the field distribution across the cross-section located at plane D. In all panels, the arrows represent the electric field vector in the considered plane, which the colormap represents the E_z-component for the quasi-BIC modes, and the E_x-component for the GMR. By analyzing the individual field profiles and their spatial distribution, we notice several notable characteristics of the resonances. Firstly and most importantly, we notice that for both classes of resonances, the optical field is largely concentrated in the nearfield of the silicon resonators and localized in the organic coating. We observe that the intensity of the optical field is highest at plane B. This characteristic ensures a high overlap factor Γ_c through a high spatial overlap between the optical mode with the active organic layer, whose refractive index is changed upon an applied driving voltage. Secondly, the field intensity decays rapidly away from the silicon nanopillars and

becomes minimal at the location of the interdigitated array of electrodes. Consequently the latter does not affect significantly the linewidth of the resonances. Thirdly, we note that both resonances are excited with x-polarized light. However, the field distribution in the high-intensity nearfield of the resonators is z-polarised for quasi-BIC and x-polarized for GMR. Consequently, an efficient operation of the electro-optic modulator on the r_{33} component is ensured by placing the contact electrodes parallel to the x-axis for the quasi-BIC and parallel to the z-axis for the GMR.

The electrodes are then employed to establish the orientation of the electro-optic tensor with respect to the geometrical coordinate system of the sample and thus the r_{33} electro-optic coefficient. r_{33} corresponds, by definition, to the direction of the electric field lines of the poling field. To visualize the latter, we provide in Fig. S3 also the electrostatic simulations of a DC field upon an applied voltage for all discussed planes A-D. We find that, as expected, the field amplitude decays far away from the electrodes and as a result, both the local r_{33} coefficient and the total introduced refractive index change Δn will be lower at plane C than at plane A. Finally, we underline two important last aspects. First, while the quasi-BIC optical mode is circulating, the GMR mode is mainly linearly polarised, thereby leading to a larger overlap factor with the r_{33} component for the GMR mode and the larger tuning of the resonant wavelength $\Delta\lambda_{res}$ for the GMR. Secondly, because of the high-Q nature of the quasi-BIC modes, an optimal height for the silicon dioxide pedestal was found experimentally to be at 300 nm (see discussion below) to ensure at the same time a narrow-band resonance and a large electro-optic tuning at given applied voltage. Increasing the pedestal height further would require higher tuning fields to achieve a commensurate effect due to the decay of the poling field as a function of height.

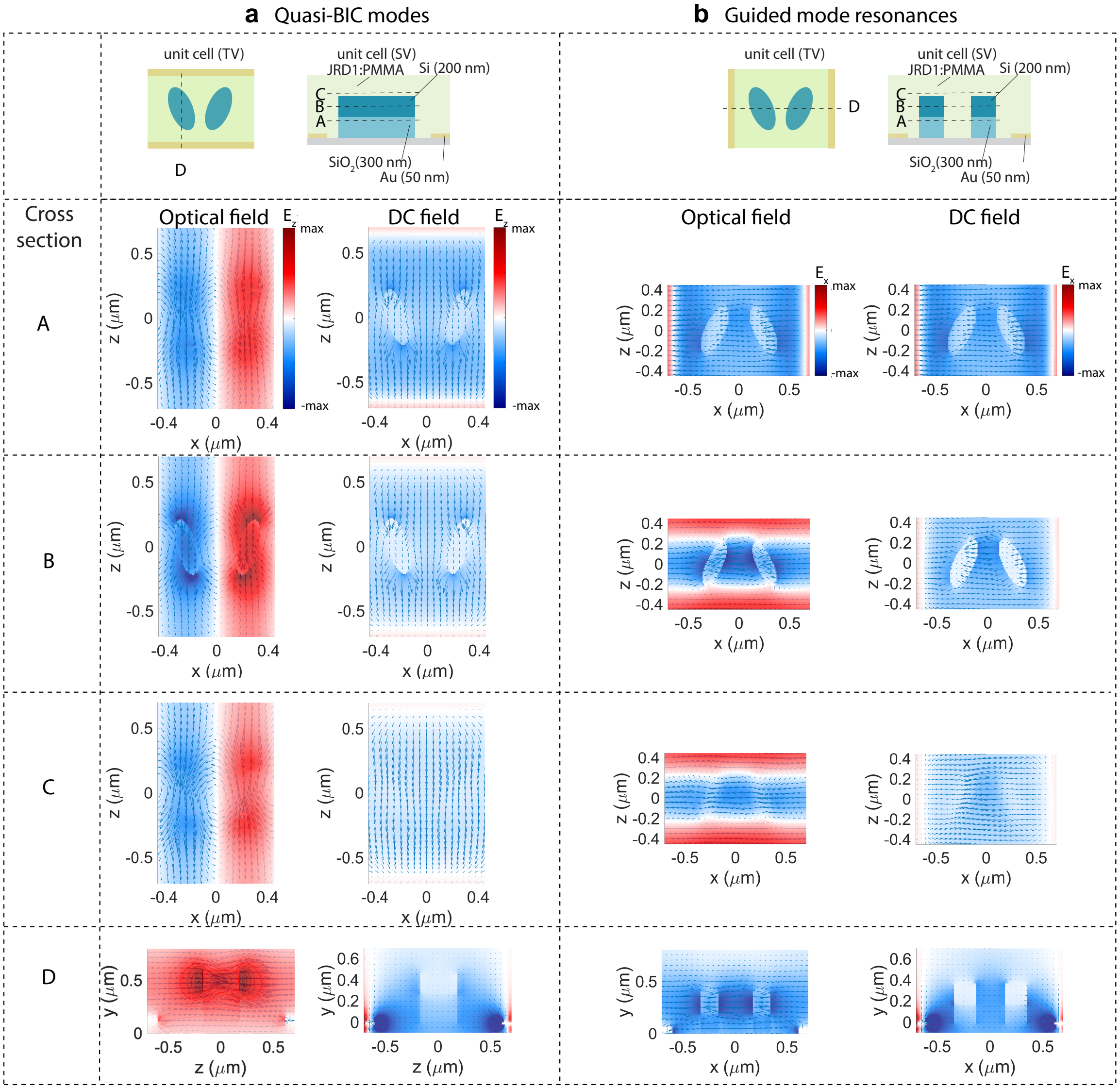

FIG. S3. **Optical and electro-static simulations.** Optical fields and DC fields of quasi-BIC modes (**a,**) and guided mode resonances (**b,**) under excitation with x-polarised light. The arrows indicate the in-plane orientation and relative magnitude of the plotted field while the colormap represents the E_z-component for quasi-BICs and E_x component for GMR (all planes A-D have the same range for the colorbar for all optical/electrostatic simulations of the quasi-BIC/GMR structures). Both classes of resonances are characterised by an optical field that is largely concentrated in the nearfield of the silicon resonators and localized in the active organic coating. The intensity of the optical field decays rapidly away from the silicon resonators. The optical mode is highly susceptible to refractive index changes in the nonlinear material, leading to the large observed tuning of its resonant wavelength. DC fields of quasi-BIC modes (**a,**) and guided mode resonances (**b,**) are indicative of the local orientation and level of alignment of the organic molecules with the poling field lines inside the three-dimensional volume of the organic layer. TV = top view, SV = side view.

B. Quasi-BICs as electro-optic modulators

In this section, we investigate the feasibility of quasi-BIC resonances ($\theta = 15°$, $\alpha = 0.725$) as free-space modulators, with a special emphasis on the losses they introduce to the incident field. For this, we compare in Fig. S4 two distinct simulations: where we consider lossless materials to the laboratory experiment where losses are taken into account. From the lossless case, we find that at resonance all light is reflected back from the metasurface and absorption is negligible at all wavelengths. In contrast to this, the real material losses introduce enhanced absorption at resonance which reduces significantly the amplitude of the reflected field. However, the visibility of the transmitted field is less impacted by the absorption. From this comparison we conclude that free-space modulators from quasi-BICs are preferably operated in a transmission configuration in the presence of losses, as we chose to do in our experiments.

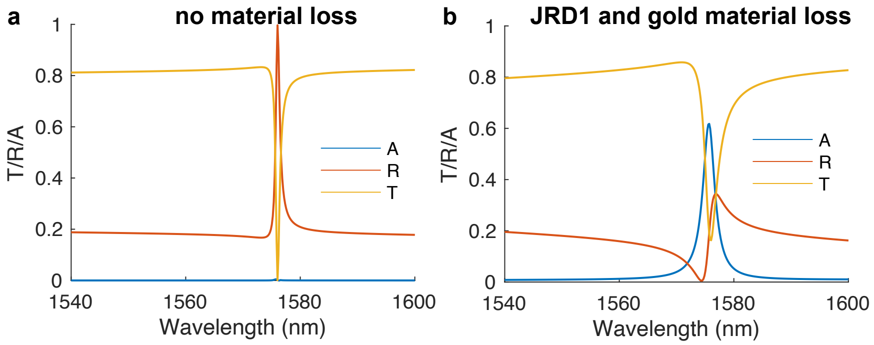

FIG. S4. **Simulated absorption, reflection and transmission curves for quasi-BIC without and with material losses. a,** Simulations when no losses are present indicate vanishingly small absorption at all wavelengths. **b,** Simulations with all material losses taken into account indicate that enhanced absorption also occurs at resonance.

C. Influence of asymmetry angle on optical linewidth

As discussed extensively in reference [2], the linewidth and the associated radiative Q-factor of the quasi-BIC resonances is directly linked with the asymmetry angle θ. In Fig. S5 we report this characteristic exemplarily for $\alpha = 0.7$. A similar behavior can be found for $\alpha = 0.725$, which is shown in Fig. S6. We find that generally, the resonant wavelength shifts towards higher wavelengths for higher asymmetry angle θ, thereby confirming our experimental results shown in the main text. In addition, at small asymmetry angles θ, the

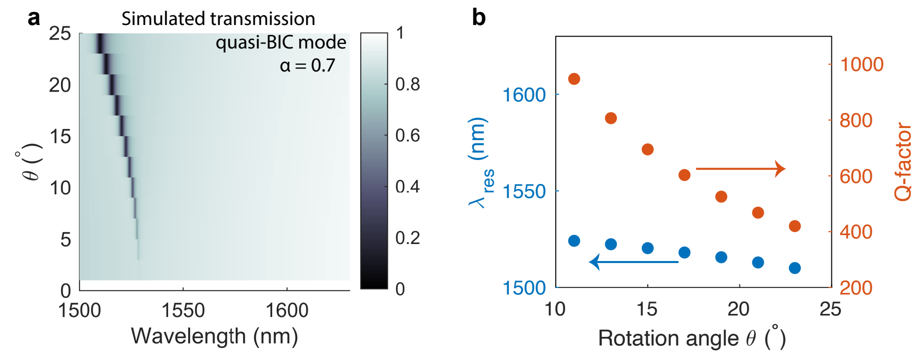

FIG. S5. **Linewidth tuning by angle θ. a, - b,** The geometrical scaling factor is $\alpha = 0.7$ and dimensions are as given in the methods. We compute the quality factor using the formula $Q = \frac{\omega_{res}}{\gamma}$ with γ the total loss rate of the resonance which we extract by fitting a Lorentzian lineshape to the transmitted power of the shape $I(\omega) = \frac{A}{(\omega-\omega_{res})^2+(\frac{\gamma}{2})^2} + B$, where A, B, ω_{res} and γ are fitting parameters. Owing to the presence of material losses introduced by the metallic electrodes and the JRD1:PMMA layer, the visibility of resonances vanishes for small θ.

visibility of the resonances is reduced owing to the material losses introduced by both the electrode materials and the JRD1:PMMA layer, as discussed in the section below.

D. Influence of conductive electrodes on optical linewidth

In this section we investigate the employed gold electrodes as a source of optical loss that both reduces the quality factor of the resonators and the visibility of the resonances. Both aspects have negative impact on the achieved overall modulation efficiency η_{max}, as they lower the amount of transmitted intensity modulation at a given applied voltage.

In Fig. S6, we compare by simulations two distinct cases: In a,-b, we depict simulation results of the optical transmission, as well as their Q-factors for an exemplarily chosen structure of $\alpha = 0.725$ that contains gold electrodes. In c,-d, we provide simulation results for identical structures where the electrode material is replaced from gold to indium tin oxide (ITO with real and imaginary refractive index as shown in e), a transparent conductive electrode that features good conductivity and much lower optical losses. From the shape of the resonance and its visibility, we compute in each case the maximum modulation depth that can be achieved with a given structure, which we define as $\Delta T_{max}/T_{max} = (T_{max} - T_{min})/T_{max}$. From our simulations, we find that replacing the electrodes by ITO is a viable path if lower optical losses are desired. In this case, both the quality factor and the visibility of optical resonances is dramatically improved. For example, a maximum modulation depth

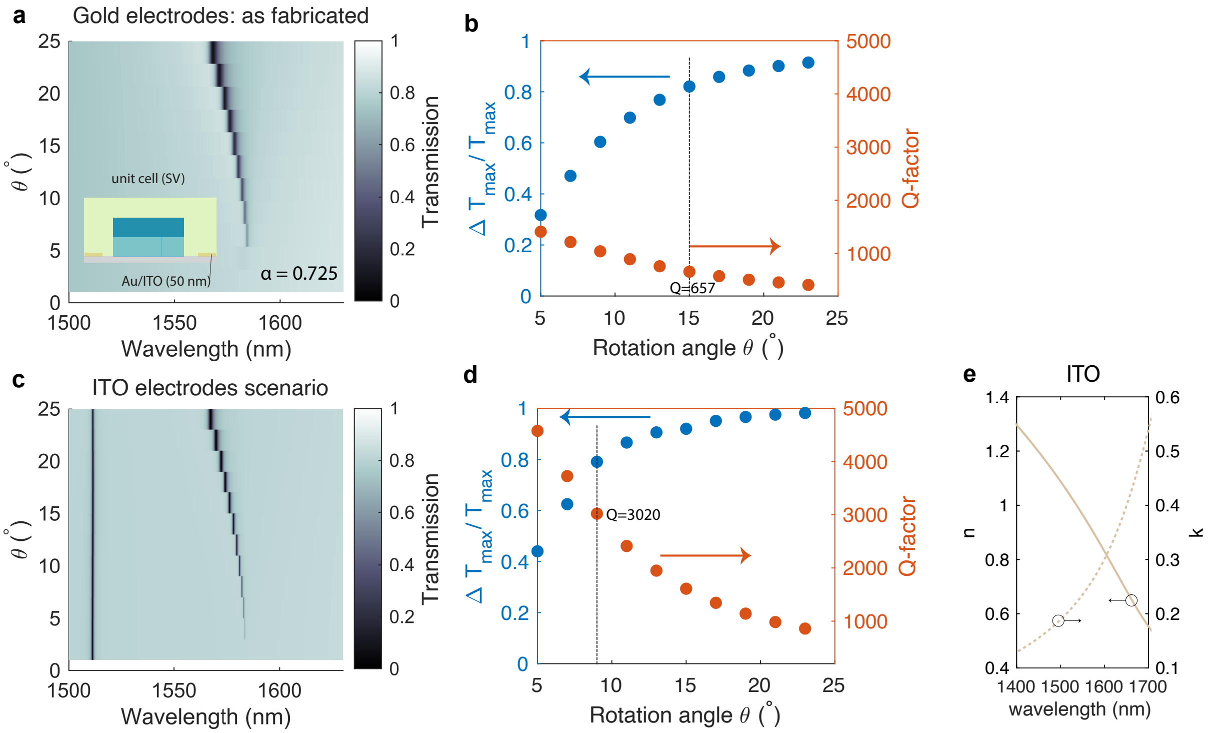

FIG. S6. **Influence of electrode material on the quality factor and the modulation capabilities of the modulators. a, - b,** Simulated resonance and modulation properties for quasi-BIC structures as-fabricated with a geometrical scaling factor of $\alpha = 0.725$ and gold-electrodes. **c, - d,** Equivalent simulations performed by replacing the gold electrodes by indium-tinn-oxide electrodes shows a considerable increase in possible quality factor, as well as the maximum modulation depth that can be achieved at given asymmetry angle. $\Delta T_{max}/T_{max} = (T_{max} - T_{min})/T_{max}$ is defined as the maximum minus the minimum of the transmission curves shown in **a, and c,** divided by the maximum of the transmission curve. **e,** Real and imaginary refractive index of ITO used for simulations.

of 0.8 is achieved in the case of gold at an asymmetry angle $\theta = 15°$, corresponding to a quality factor of Q = 657, whereas the same modulation depth is achieved in the case of ITO at an asymmetry angle $\theta = 9°$, corresponding to a quality factor of Q = 3020, roughly 5 times higher.

Furthermore, it is important to note that replacing the electrode material by ITO also improves the extinction ratio of the modulators. If the simulated extinction ratio at $\theta = 25°$ is 10 dB for gold electrodes, it reaches 19 dB for ITO electrodes.

E. Influence of pedestal height on optical linewidth

We show in the section above that the optical fields are well concentrated around the silicon nanostructures at resonance. For the case of electrically actuated structures as the ones discussed here, electrodes are mandatorily incorporated with the nanostructures to provide the necessary actuating fields. While previous proposals may choose to apply electrodes that enclose several periods of the nanoresonator array as a way to keep the metallic losses low, we chose to apply a set of electrodes to each single row of the array. This configuration notably maximized the electro-optic effect at a given voltage, which scales linearly with the electric field. However, in this case, as electrodes are present in the immediate vicinity of the nanostructures, losses introduced by the metallic electrodes can become significant, if no additional measures are taken.

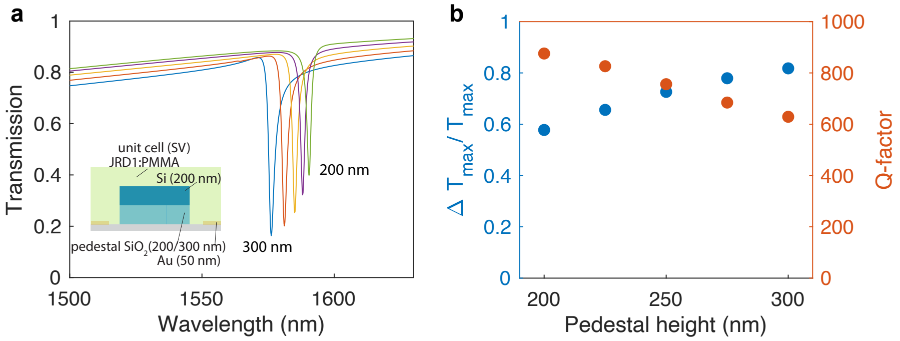

FIG. S7. **Influence of pedestal height on the quality factor and the modulation capabilities of the modulators. a,** Simulated resonance for quasi-BIC structures as-fabricated with a geometrical scaling factor of $\alpha = 0.725$ and gold-electrodes, for different heights of the silicon dioxide pedestal from 200 nm to 300 nm. The visibility of the resonance decreases rapidly with decreasing pedestal height. **b,** Quality factor and modulation efficiency for different pedestal heights. $\Delta T_{max}/T_{max} = (T_{max} - T_{min})/T_{max}$ is defined as the maximum minus the minimum of the transmission curves shown in **a,** divided by the maximum of the transmission curve.

In our case, we chose to place the silicon nanoresonators on top of silicon dioxide pedestals that are used as a spacer to allow a placement of the electrodes further away from the high-intensity regions of the nearfield. As a result, the choice of the height of this pedestal is extremely important, and is analysed in this section. In Fig. S7 we show exemplarily the effect of the height of the pedestal on the visibility and the modulation efficiency of free-space modulators based on quasi-BICs with an $\alpha = 0.725$. Clearly, for decreased pedestal heights, the visibility of the resonance decreases as well as the modulation efficiency.

F. Influence of electrode height on resonance

The thin electrodes and in particular their physical localization below the silicon nanoresonators leads to a distribution of the DC/RF tuning field that is not uniformly parallel to the z-axis. This fact is visible e.g. in the electric field distribution plots we provided in Fig. S3. One possibility to improve this alignment can be to increase the electrode thickness to become commensurate with the silicon resonators themselves. Unfortunately, in our geometry, this concomitantly introduces significant optical losses due to a higher overlap of the BIC mode with the electrode material, thereby lowering the quality factor of the resonance considerably. We demonstrate the impact of the electrode height on the linewidth and the depth of the resonance by simulations for two independent scenarios in Fig. S8: where the electrodes are from gold or from ITO. As a result, both the intensity modulation and the modulation per volt is expected to significantly deteriorate when the electrodes are introducing too significant losses.

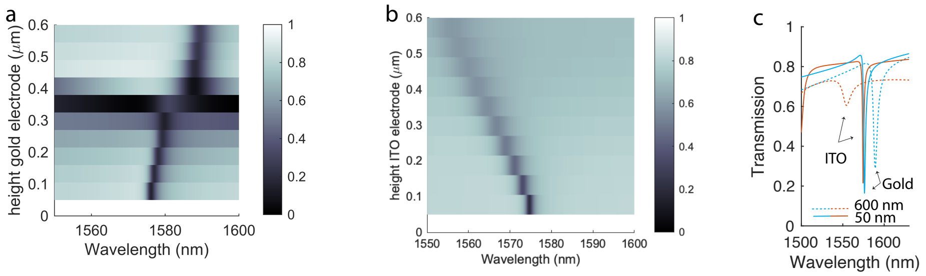

FIG. S8. **Influence of electrode height on quality factor and resonant depth of quasi-BICs. a,** Gold electrodes. **b,** ITO electrodes. **c,** Comparison of the two for two different electrode heights (50 and 600 nm).

G. Influence of incident angle on resonance and modulation depth

Here we analize the angle-dependent resonance of quasi-BICs and the introduced modulation depth by measurements and simulations of a representative sample with $\alpha = 0.7$, $\theta = 15°$. We show in Fig. S9 below first the angular dependency of the optical resonances as measured by experiments and simulated using CST microwave studio. We find that an incident angle that deviates from the normal broadens the resonance and lowers its visibility and that the resonant wavelength shift stronger when the tilt is oriented as shown in Fig. S9 b and d. Our simulations agree very well with the measurements.

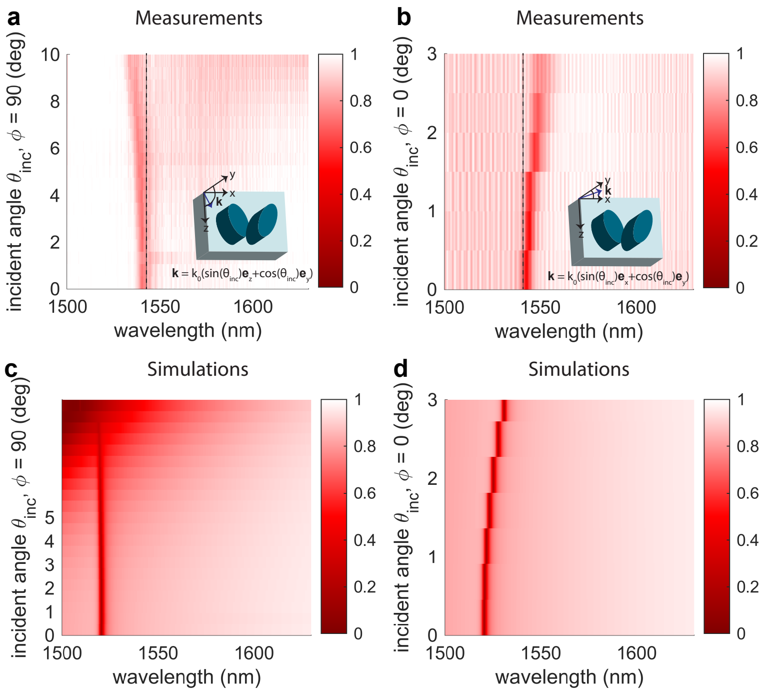

FIG. S9. **Angle-dependent resonance transmission of quasi-BIC are compared in measurements and simulations. a,-b,** Measurements. **c,-d,** Simulations.

In addition, we investigate in Fig. S10 the dependency of the modulation depth as a function of incident angle while the device is operated as an electro-optic transducer. In a first set of measurements, we report the resonance shift and the change in modulation as a function of the incident angle θ_{inc} when $\Phi = 0°$. At zero applied bias (Fig. S10 b), we observe a red-shift of the resonance that exceeds its linewidth around an angle of $\theta_{inc} = 2°$. This angle-dependence is clearly distinct from the behavior of GMR which rather split into two modes [1] at incident angles different from $\theta_{inc} = 0°$. In Fig. S10 c, we report the normalized intensity modulation upon an applied $V_{RF} = 20V \times \sin 2\pi 1MHzt$ and identify, as expected, that also the modulation red-shifts and changes magnitude with incident angle. In Fig. S10 d, we plot the intensity modulation, as well as the normalized intensity modulation for two exemplary operation wavelengths, marked by the blue and red lines. We find that the strength of the intensity modulation depends on the chosen operation wavelength (e.g. can switch sign by operating on the falling or rising flanks of the resonance and is shallow away from the resonance) and that it does not decay below 3 dB over an angular range of $\theta_{inc} = \pm 1°$. Quite on the contrary, the modulation efficiency is much less affected when changing the incident angle in the orthogonal plane (when $\Phi = 90°$), as shown in Fig.

Fig. S10 e-g. In this case, we find that the modulation intensity remains relatively flat over an angle of $\theta_{inc} = \pm 4°$.

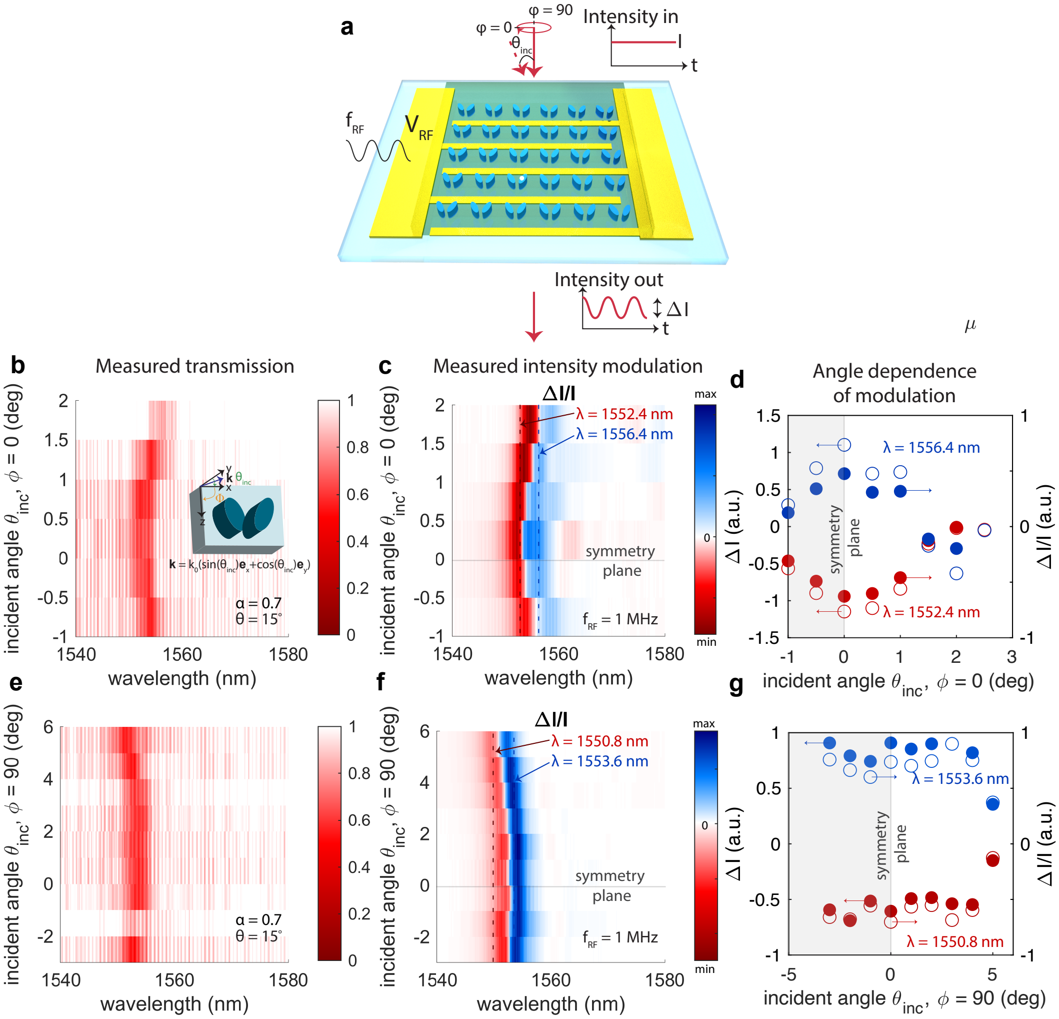

FIG. S10. **Angle-dependent resonance transmission and modulation depth of quasi-BIC. a,** Geometry. **b,-d,** Resonance shift and the change in modulation as a function of the incident angle θ_{inc} when $\Phi = 0°$ **e,-g,** Resonance shift and the change in modulation as a function of the incident angle θ_{inc} when $\Phi = 90°$.

S4. A 3 × 3 SPATIAL LIGHT MODULATOR

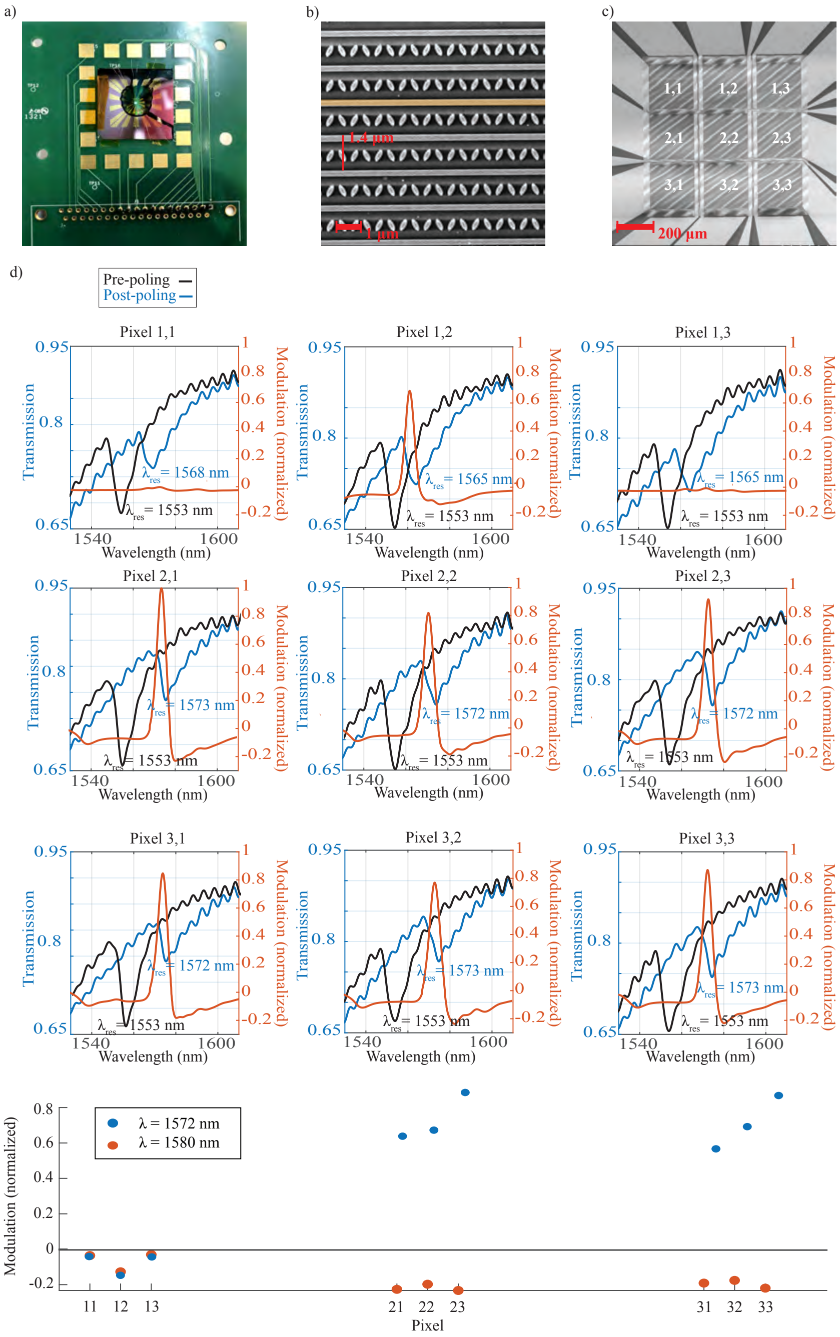

FIG. S11. **A 3 × 3 spatial light modulator realized from quasi-bound states in the continuum. a,** Camera picture of the SLM mounted on a PCB that provides the necessary bonding pads to multiplex the driving voltages of the different pixels. **b,-c,** Scanning electron microscope pictures of the employed structures for the quasi-bound states in the continuum resonances and the entire array featuring a totality of 9 pixels. **d,** Optical resonance properties of each pixel are provided prior to poling (black) and after poling (blue) together with normalized wavelength-resolved electro-optic modulation curves. We observe that two out of nine pixels are not electro-optically active (1,1 and 1,3) and that one has an optical resonance that is located elsewhere from the rest of the pixels (1,2). **e,** Modulation is compared for all pixels at two exemplarily chosen operation wavelengths.

We demonstrate the ability to modulate an optical beam in the two-dimensional plane by arranging a total of 9 parallel pixels into one single 3×3 spatial light modulator (SLM), following a similar strategy as first demonstrated here [1]. After fabrication of the spatial light modulator, all 9 pixels are activated by electric field poling in parallel, in one single run, by contacting all of them via electrical wire bonds in parallel.

To drive the spatial light modulator, we mount the fabricated sample on a printed circuit board that provides the electrical contact pads. The PCB has a hole of diameter 1 cm such that the spatial light modulator can be operated in transmission. To characterize the samples, we use a voltage driver from National Instruments to drive the entire chip, as shown in Fig. S11 a. A close-up scanning electron micrograph figure of the fabricated structure is provided in Fig. S11 b, and a zommed-out figure in Fig. S11c.

In Fig. S11 d we provide experimental data of the fabricated spatial light modulator. We show in black the optical resonance of each pixel prior to poling and find that all different pixels have consistent resonance conditions around $\lambda_{res} = 1553$ nm. In blue we then provide the optical resonance spectrum of each pixel after poling. We find that the entire upper row of pixels has clearly distinct resonances compared to the resonances of the two lower rows of pixels, which are centered around $\lambda_{res} = 1572$ nm. This result already points into the direction, that the poling procedure may not have been equally efficient in the upper row compared to the two lower rows. We attribute this effect to possible local leakage channels within each structure. In orange we then report the wavelength-resolved electro-optic modulation of each pixel. For a facilitated comparison, we normalize all curves to the maximum modulation we measure for all pixels, which is in this case found for pixel 2,1. We find that two pixels in the upper row do not exhibit modulation, hinting to the possibility that the poling procedure was not successful for these two pixels on this particular sample. In the two lower rows, the normalized modulation varies between 0.8 and 1. In Fig. S11 e, we plot the measured normalized modulation for two exemplarily chosen operation wavelengths. While the modulation amplitude may change within a range of up to approximately 25%, this effect may be accounted for by prior calibration of each pixel of the spatial light modulator and subsequent adequate application of driving voltage to effect an uniform modulation across the entire structure.

S5. PERFORMANCE COMPARISON WITH SIMILAR ELECTRO-OPTIC META-SURFACE STRUCTURES

Our work demonstrates a brand-new material platform consisting of hybrid silicon-organic flat optical structures from Mie resonators, that exhibits, to the best of our knowledge, superior key figures of merit over current state of the art across various electro-optic material platforms even when integrated with GHz electrodes. We compare our demonstration to previously reported structures in the table below. We note that not only the employed material systems are different among all compared structures but also the explored optical resonances and wavelength of operation. Consequently, generally speaking, all material systems may leverage novel resonances for improved performance in the future.

Fig. of merit/platform	Our demonstration	Lithium niobate [3]	Barium titanate [4]	Improvement our work
Abs. transm./refl. mod.	0.3 @100 V	0.0001 (0.01%) @10 V	0.001 (0.1%) @4 V	300 – 3000 ×
Abs. transm./refl. mod. per V	0.3/100 V = 0.003 V^{-1}	0.00001 V^{-1}	0.00025 V^{-1}	10 – 300 ×
Maximum mod. speed	5 GHz, 3 GHz 3 dB	Up to 2.5 MHz	20 MHz	100 – 1000 ×
Maximal refr. index change	0.04	Not specified	9.9×10^{-4}	40 ×

TABLE S1. **Comparison of state-of-the-art free-space electro-optic platforms from $\chi^{(2)}$ Pockels materials**: Figures of merit of our demonstration compared to reported state-of the art electro-optic free-space modulators based on Pockels effect. Clearly, our platform has a performance that is at least one, if not several orders of magnitude better.

In our specific implementation, we demonstrate a modulation of the absolute transmission $\Delta T = 0.6$ by applying a voltage swing from $-100\ V$ to $100\ V$. We note that this already demonstrates the capability of our structures to - in principle - sustain such large voltages. A detailed analysis of the on-off-switching behavior of the material system is provided in reference [1]. Consequently, owing to both the large built-in electro-optic coefficient of the structure, the large overlap factors of the employed quasi-bound states in the continuum, their narrow linewidth and the large applied voltage, the achieved absolute transmission modulation is two to three orders of magnitude higher than previously reported free-space electro-optic modulators. The effect of the large applied voltage may be accounted for by investigating the absolute transmission modulation per applied volt, which in our case amounts to 0.003 V^{-1}. Also here, our platform performs at least one order of magnitude better. This stems from the optimized electro-optic effect that employs both an in-plane periodically poled film of JRD1:PMMA and that we apply the voltage across single rows

of sub-wavelength resonators rather than several rows as e.g. in ref. [3]. Furthermore, we demonstrate experimentally an efficient electro-optic modulation up to 5 GHz, with a 3 dB electro-optic bandwidth of 3 GHz, by incorporating the metasurfaces with microwave coplanar waveguides. We characterize the electro-optic response in detail and link the current cut-off to the RC-time constant of the interdigitated electrode array. We note that other electro-optic material systems may adopt a similar strategy and thereby reach higher modulation speeds than currently demonstrated. Finally, the maximal refractive index change achieved of $\Delta n = 0.04$ is considerably increased over current state of the art due to a high electro-optic coefficient of the employed material system and a high built-in driving electric field that is supported by the structure.

A. Possible routes to decreasing the switching voltage and increasing the modulation strength

We have shown in the previous sections that several geometrical and material parameters influence directly the linewidth of the employed optical resonances and, therefore, also the modulation efficiency that can be achieved. Although we show above that our platform shows significant improvement of the modulation performance over other demonstrated state-of-the art platforms, it is highly desirable to decrease the driving voltage that is necessary to achieve full modulation. An ideal milestone will be reached when such structures will provide full intensity modulation already at CMOS-compatible voltages. While achieving this falls outside the scope of the current study, we outline here several approaches that may be taken towards reaching that goal.

A first possible approach would be to use a different electrode material than gold, such as ITO, which minimized the optical losses and therefore enables higher-Q resonances. We show in section S3 D that operating on an asymmetry angle $\theta = 9°$ in conjunction with ITO leads to an improvement of the quality factor by an approximately a factor of 5 compared to operating on an asymmetry angle $\theta = 15°$ in conjuction with gold electrodes. In both cases, the modulation depth is nearly identical. This approach would then lead to a decrease of the switching voltage from $V_{switch} = 60$ V, as demonstrated experimentally in the main text Fig. 3 d-f to $V_{switch} = 12$ V.

A second approach that may be followed in the future would be the placement of a

second set of electrodes on the top of the structure, patterned symmetrically around the silicon electrodes, as shown in Fig. S12 below (color plot represents magnitude of Ez and arrows the in-device DC field). By connecting the top pair of electrodes to the same voltage source as the bottom pair, a symmetric field distribution can be achieved. We demonstrate this concept by simulations and show below the in-device DC field for the two scenarios (Fig. S12 a: single electrode pair and Fig. S12 b: double electrode pair, applied voltages are marked in the colorplots). In Fig. S12 c we compare the Ez field at the center of the silicon nanoresonators (marked by the dotted lines) and find the expected enhancement of the built-in field by a factor of 2 when using two electrode pairs.

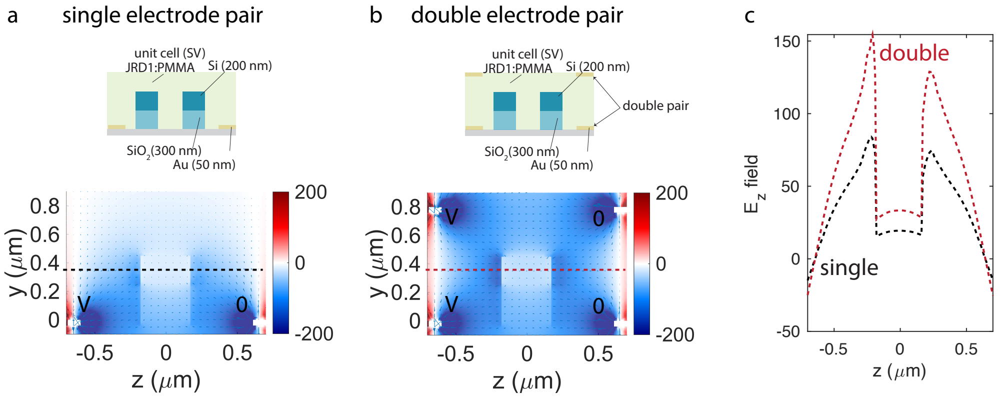

FIG. S12. **Single versus double pair of electrodes. a,** In-device electric field when one single electrode pair is used. **b,** In-device electric field when two electrode pairs are used. **c,** Comparison of the built-in field for the two cases at the location of the dashed line.

Further improvement of the switching voltage may be achieved by optimizing the poling procedure of the organic electro-optic layer towards their maximum reported electro-optic coefficient of 560 pm/V, e.g. by optimizing the mixing ratio between PMMA and JRD1. Finally, a vertical architecture where ITO electrodes are placed on top and on the bottom of the structure instead of laterally may provide a way to lower the applied voltage for a given built-in field as well as ensure a homogenous distribution of the electro-optic refractive index change across the entire structure. All of these proposals require in-depth studies of the optical, electronic and electro-optic properties of the resulting structures and go beyond

the scope of this study.

---

[1] Ileana-Cristina Benea-Chelmus, Maryna Meretska, Delwin L. Elder, Michele Tamagnone, Larry R. Dalton, and Federico Capasso, "Electro-optic spatial light modulator from an engineered organic layer," Nature Communications **12**, 5928 (2021).

[2] Kirill Koshelev, Sergey Lepeshov, Mingkai Liu, Andrey Bogdanov, and Yuri Kivshar, "Asymmetric Metasurfaces with High- Q Resonances Governed by Bound States in the Continuum," Physical Review Letters **121**, 193903 (2018), arXiv:1809.00330.

[3] Helena Weigand, Viola V. Vogler-Neuling, Marc Reig Escalé, David Pohl, Felix Richter, Artemios Karvounis, Flavia Timpu, and Rachel Grange, "Enhanced electro-optic modulation in resonant metasurfaces of lithium niobate," ACS Photonics **8**, 3004–3009 (2021).

[4] Artemios Karvounis, Viola V. Vogler-Neuling, Felix U Richter, Eric Dénervaud, Maria Timofeeva, and Rachel Grange, "Electro-Optic Metasurfaces Based on Barium Titanate Nanoparticle Films," Advanced Optical Materials **8**, 2000623 (2020).
